# Association between the -112G/A polymorphism of uteroglobulin-related protein 1 gene and asthma risk: A meta-analysis

**DOI:** 10.3892/etm.2014.1471

**Published:** 2014-01-02

**Authors:** HAOJUN XIE, MULI WU, BIN SHEN, YI NIU, YATING HUO, YUANXIONG CHENG

**Affiliations:** 1 Department of Respiratory and Critical Care Medicine, Nanfang Hospital, Southern Medical University, Guangzhou, Guangdong 510515, P.R. China; 2 Department of Cardiology, Nanfang Hospital, Southern Medical University, Guangzhou, Guangdong 510515, P.R. China

**Keywords:** uteroglobulin-related protein 1 gene, asthma, polymorphism, meta-analysis

## Abstract

The aim of this study was to investigate the correlation between the -112G/A polymorphism of the uteroglobulin-related protein 1 (UGRP1) gene and asthma risk using meta-analysis. PubMed, BIOSIS Previews and EBSCOhost were searched, and data were extracted independently by two reviewers. Odds ratios (ORs) with corresponding 95% confidence intervals (CIs) were used to assess the strength of the associations. Statistical analysis was performed using Review Manager 5.2 and STATA 11.0 software. Six studies, involving 816 cases and 1,165 controls, were included in the analysis. The meta-analysis showed a significant correlation between the UGRP1-112G/A polymorphism and asthma for AA versus GG (P=0.01) and AA versus GA/GG (P=0.02). Furthermore, stratification by ethnicity revealed a significant correlation between the -112G/A polymorphism and asthma for A versus G (P=0.02), AA versus GG (P=0.01) and AA versus GA/GG (P=0.03) in Asians, but not in Caucasians. When stratified by atopy, a significant correlation was observed for A versus G (P=0.02) and AA/GA versus GG (P=0.04) in the mixed group. No correlation was observed for age stratification. Results of the current meta-analysis indicate that the -112G/A polymorphism of the UGRP1 gene is likely to contribute to asthma risk, particularly in the Asian population.

## Introduction

Asthma is a common global public health concern, affecting ~300 million people worldwide (1). It is a complex disease, which is the result of interactions between genetic and environmental factors (2). Investigation of the correlation between genetic variants and asthma risk has identified numerous genes conferring susceptibility to asthma (3); among these, the uteroglobulin-related protein 1 (UGRP1) gene has been extensively studied.

The gene encoding UGRP1, a secreted protein, was first identified by Niimi *et al* (4). The human UGRP1 gene is located on chromosome 5q31–32, a region containing a number of candidate genes that may play a role in asthma and other allergic diseases. These genes encode proinflammatory cytokines, such as interleukin-3, −4, −5, −9 and −13 (5). As a result of the similarity in the amino acid sequences of UGRP1 and Clara cell protein (CC16), which exhibits several immunomodulatory and anti-inflammatory effects, it is possible that UGRP1 may possess similar functions (6). Furthermore, UGRP1 mRNA is predominantly expressed in the lung, with a high level of expression in the epithelial cells of the lung airway (4). These observations suggest that the UGRP1 gene may be important in the pathogenesis of asthma.

Previous studies have investigated the association between the -112G/A polymorphisms of the UGRP1 gene and asthma risk. However, while certain studies have described a significant association (4,7), no such association was observed in other studies (8–12). Since a single study may lack the robust power to provide a reliable conclusion, in the present study, a comprehensive search of the literature and a meta-analysis was performed to examine whether UGRP1 gene polymorphisms contribute to asthma susceptibility.

## Materials and methods

### Publication search

Pubmed, BIOSIS Previews and EBSCOhost were comprehensively searched, with the last search updated on March 12, 2013. The Medical Subject Heading terms and/or text words utilized were ‘asthma’ or ‘bronchial hyperreactivity’ or ‘respiratory hypersensitivity’ or ‘bronchial asthma’, in combination with ‘polymorphism*’ or ‘variant*’ or ‘genetic’ or ‘mutant*’ and in combination with ‘SCGB3A2’ (secretoglobin, family 3A, member 2) or ‘UGRP1’ or ‘Clara cell secretory protein-related protein’. No publication language restrictions were imposed. All the searched studies were retrieved, and their references were checked for other relevant publications. The search strategy for the study is shown in Table I.

### Inclusion and exclusion criteria

Human studies were included if they met the following criteria: i) Evaluation of the -112G/A polymorphism of the UGRP1 gene and asthma risk; ii) using a case-control design; and iii) genotype distributions in comparison groups were available for estimating an odds ratio (OR) with 95% confidence interval (CI). Studies were excluded if one of the following existed: i) Not relevant to UGRP1 gene polymorphisms or asthma risk; ii) design based on family or sibling pairs; and iii) reviews or abstracts. When the same patient population was included in several publications, only the most complete study was included in the meta-analysis. If the original data for the genotype frequencies were unavailable in the relevant studies, an email was sent to the corresponding author for additional data.

### Data extraction

Two investigators (Xie and Wu) independently reviewed the full manuscripts of the eligible studies, and data were extracted independently into a predesigned data collection form. The accuracy of the data was verified by comparing the collection forms from each investigator. Disagreements were resolved by discussion or by a third author (Cheng) assessing the articles. The following information was collected from each study: First author’s name, year of publication, original country, ethnicity, sample size, asthma definition, genotyping method, atopic status and genotype numbers in the cases and controls.

### Quality score evaluation

The quality score evaluation was performed in accordance with a previous study (13). Briefly, the following variables were assessed: Representativeness of cases and controls, ascertainment of asthma and controls, genotyping examination, Hardy-Weinberg equilibrium (HWE), association assessment and response rate. The quality score had a maximum of 15 points. The higher the study scored, the better the quality was. Studies with quality scores <4 were excluded (14). All studies included in our meta-analysis were of a high quality (Table II).

### Publication bias

The publication bias of the studies was assessed using Begg’s funnel plots, and P<0.05 was considered to indicate a statistically significant difference. Since this method required a range of studies with varying sizes and subjective judgments (14), publication bias was also evaluated using Egger’s linear regression test.

### Statistical analysis

Departures from the HWE in the control groups were assessed using the χ^2^ test. The meta-analyses were performed using the following models: i) Allelic (A versus G); ii) additive (AA versus GG); iii) recessive (AA/AG versus GG); and iv) dominant (AA versus AG/GG). Subgroup analyses were conducted according to ethnicity, age and atopic status. The heterogeneity between the studies was assessed using the χ^2^ test, based on the Cochrane Q-test. In addition, I^2^ was used to examine the heterogeneity among the included studies. P>0.10 for the Q-test indicated a lack of heterogeneity among the studies. The pooled OR estimate of each study was then calculated using the fixed effects model. Otherwise, the random effects model was used.

All statistical tests were performed using Review Manager software (version 5.2; The Nordic Cochrane Center, Copenhagen, Denmark) and STATA 11.0 software (Stata Corp., College Station, TX, USA). P<0.05 was considered to indicate a statistically significant difference, with the exception of heterogeneity tests where a level of 0.10 was used.

## Results

### Studies included in the meta-analysis

**Figure 1:**
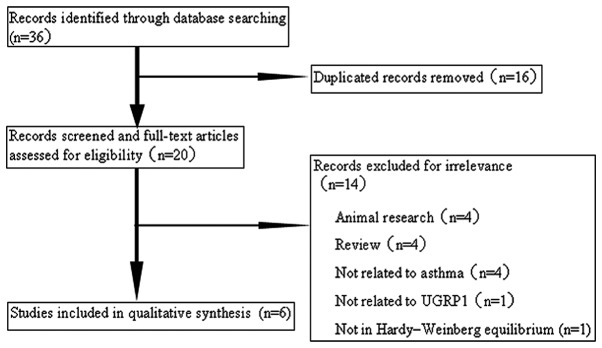
Flow of study identification, inclusion and exclusion. UGRP1, uteroglobin-related protein 1.

Fig. 1 outlines the selection process. Briefly, a total of 36 articles were identified in the initial search. Having reviewed the titles, abstracts and full-texts, and removed the duplications, six relevant articles were included in the meta-analysis. These eligible case-control studies contained 816 cases and 1,165 controls. Two studies investigated a Caucasian population and four investigated an Asian population. Three studies were performed with adults and three with children. Two studies included only patients with atopic asthma, two studies included patients with atopic asthma and those with non-atopic asthma (data for these patients were able to be separately extracted) and two studies did not offer detailed information with regard to atopic status. The characteristics of each study included in the meta-analysis are presented in Table II.

### Meta-analysis of the UGRP1 gene -112G/A polymorphism and asthma

**Figure 2:**
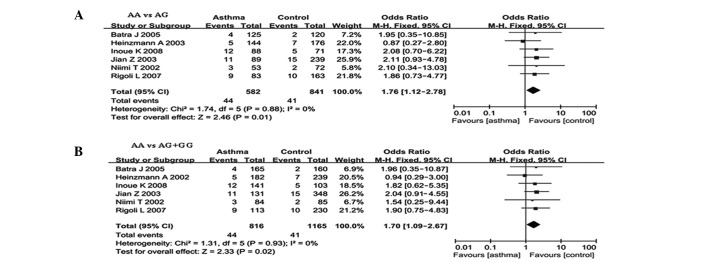
ORs and 95% CIs of individual and pooled results for the association between the UGRP -112G/A polymorphism and asthma for all the studies. (A) AA vs. GG; (B) AA vs. GA/GG. OR, odds ratio; CI, confidence interval; UGRP-1, uteroglobin-related protein 1.

**Figure 3:**
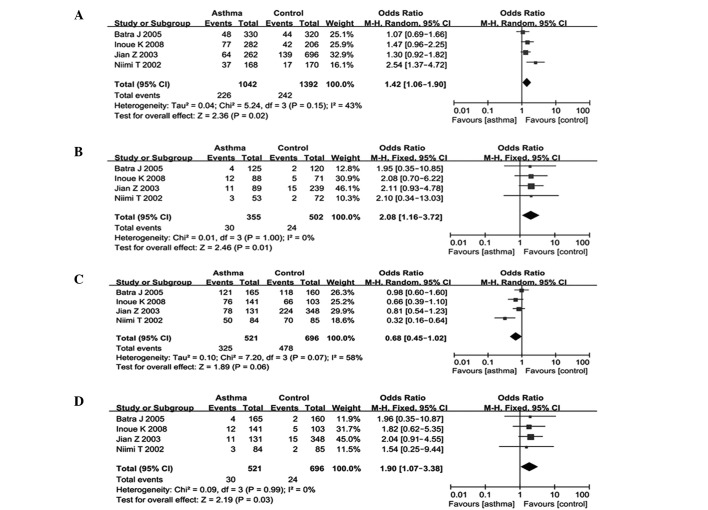
ORs and 95% CIs of individual and pooled results for the association between the UGRP -112G/A polymorphism and asthma for the Asian subgroup. (A) A vs. G; (B) AA vs. GG; (C) AA/GA vs. GG; (D) AA vs. GA/GG. OR, odds ratio; CI, confidence interval; UGRP-1, uteroglobin-related protein 1.

The meta-analysis results are shown in Table III. The combined results of all the studies showed that there were significant associations between the UGRP1-112G/A polymorphism and asthma risk in the genetic model of AA versus GG (OR, 1.76; 95% CI, 1.12–2.78; P=0.01) and in the genetic model of AA versus GA/GG (OR, 1.70; 95% CI, 1.09–2.67; P=0.02) (Table III and Fig. 2). In the subgroup analysis by ethnicity, significant associations were observed among Asians in the genetic model of A versus G (OR, 1.42; 95% CI, 1.06–1.90; P=0.02), AA versus GG (OR, 2.08; 95% CI, 1.16–3.72; P=0.01) and AA versus GA/GG (OR, 1.90; 95% CI, 1.07–3.38; P=0.03); however, these associations were not observed in Caucasian populations (Table III and Fig. 3). The subgroup analysis by atopic status showed associations for A versus G (OR, 1.84; 95% CI, 1.08–3.13; P=0.02) and AA/GA versus GG (OR, 0.47; 95% CI, 0.23–0.97; P=0.04) in the mixed atopic group (Table III). Subgroup analysis was also performed by age, but no associations were found.

### Heterogeneity analysis

**Figure 4:**
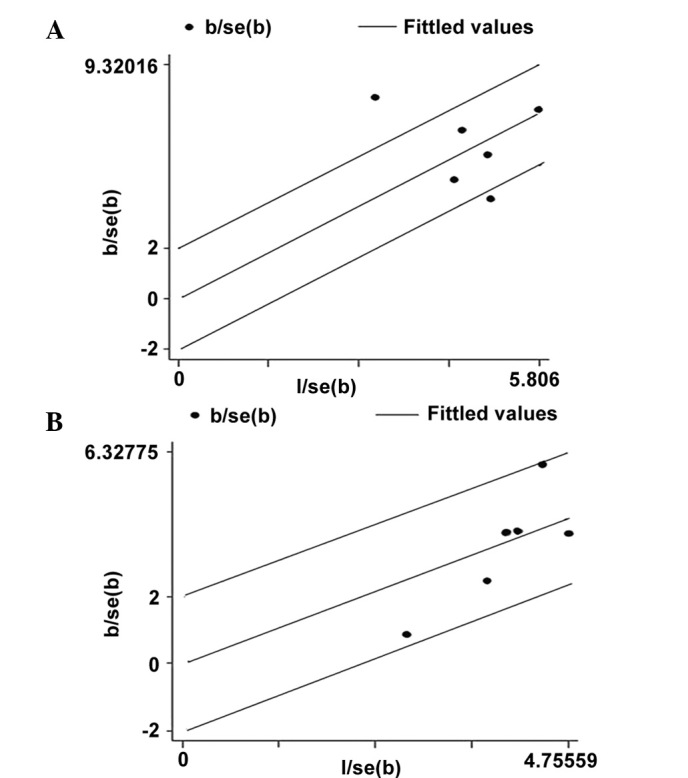
Galbraith plots of the association between the -112G/A polymorphism and asthma in the allelic and recessive models. (A) Allelic model (A vs. G). The outlying dot indicates the study of Niimi *et al*, 2002 (5). (B) Recessive model (AA+GA vs. G/G). se, standard error.

No heterogeneity was observed in the additive and dominant models, however, marked heterogeneity existed in the allelic and recessive models. Therefore, Galbraith plots were used to graphically evaluate the source of the heterogeneity. For the allelic model, one study (5) was hypothesized to be the main contributor to the heterogeneity (Fig. 4).

### Publication bias

**Figure 5:**
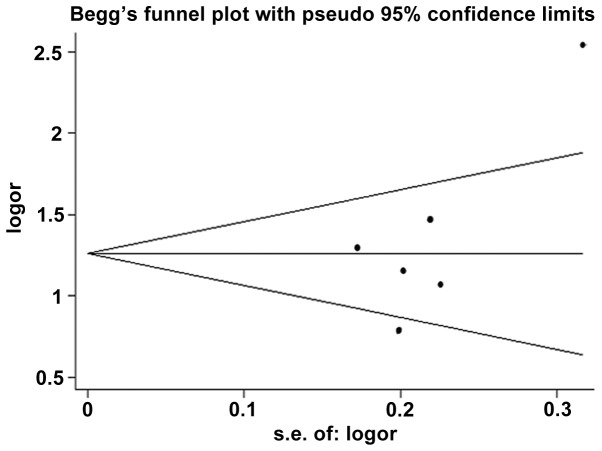
Association between the -112G/A polymorphism and asthma in all the studies for the allelic model (A vs. G). se, standard error; or, odds ratio.

Begg’s funnel plots were used to investigate the potential publication bias of the studies. The funnel plot demonstrated evidence of asymmetry for the allelic model (Fig. 5). No evidence of publication bias was observed in other comparison models using Begg’s funnel plots. In addition, no publication bias was revealed among the studies when using Egger’s regression test (P=0.109, 0.794, 0.153 and 0.482 for the allelic, additive, recessive and dominant models, respectively).

## Discussion

Asthma is a complex pulmonary disorder that is caused by numerous genetic and environmental factors and is the result of genetic and environmental interaction. The hallmarks of asthma are airway inflammation, remodeling and hyperresponsiveness (2,15). The UGRP1 gene was identified by Niimi *et al* (4), and is located in a chromosomal region harboring a number of genes involved in allergic diseases. UGRP1 is similar to CC16 with regard to its amino acid sequence and site of tissue-specific expression (4). As demonstrated by *in vivo* and *in vitro* studies (6,16), CC16 functions as an anti-inflammatory agent. Similarly, UGRP1 has been suggested to exhibit anti-inflammatory functions. It has been shown that UGRP1 is associated with an increased risk for Graves’ disease (17,18). However, there have been confounding results with regard to the correlation between UGRP1 gene polymorphisms and asthma risk (5,7,9,19). Therefore, in the present study, a comprehensive meta-analysis was conducted to examine the association between the UGRP1 gene -112G/A polymorphism and asthma risk.

The present meta-analysis of six articles, including 816 patients with asthma and 1,165 controls, investigated the association between the UGRP1-112G/A polymorphism and asthma risk. Overall, the pooled results revealed significant associations between the UGRP1-112G/A polymorphism and asthma risk in the genetic models of AA versus GG and AA versus GA/GG. To conduct a more comprehensive study of the correlation between the UGRP1-112G/A polymorphism and asthma, subgroup analyses were performed. In the subgroup analysis by ethnicity, significant associations with asthma were revealed among Asians in the allelic, additive and dominant genetic models. The allelic model suggested that carriers of the A allele, including the AA and AG genotypes, were at a 1.42-fold higher risk of asthma than G allele carriers. The additive model demonstrated that the AA genotype increased the risk by 108%, indicating that individuals with the homozygous AA genotype were likely to have a higher risk of asthma than those with a GG genotype. Furthermore, the dominant model indicated that individuals with a homozygous AA genotype were likely to have higher risk of asthma than those with AG and GG genotypes (OR=1.90). However, these associations were not observed in the Caucasian subgroup. The ethnic differences may have been due to chance, since studies with small sample sizes are likely to have a low statistical power to detect slight effects. In the stratification by atopy, associations were observed for the allelic and recessive models in the mixed group. No significant associations were revealed for the age stratification.

In the present meta-analysis, heterogeneity existed in the allelic and recessive models. This issue may have affected the interpretation of the results. In order to explore the source of heterogeneity, Galbraith plots were produced for all of the studies. As shown in Fig. 4, it is possible that one study (5) may have been the main source of heterogeneity. The funnel plots were symmetrical, except the allelic genetic model, and it was indicated that there was no significant publication bias among the selected studies using Egger’s test. Despite this, the meta-analysis results of the present study should be interpreted with caution, due to the following limitations: i) The number of studies included in the meta-analysis was small, with only two studies performed with a Caucasian population; ii) heterogeneity may have affected the meta-analysis; iii) although Egger’s regression test was performed, publication bias may still have affected the analysis, as studies with negative results may not have been published; and iv) the meta-analysis was not able to assess gene-gene and gene-environment interactions.

To the best of our knowledge, this is the first meta-analysis conducted to explore the association between the UGRP1-112G/A polymorphism and asthma risk. All studies included in this meta-analysis were of a high quality, which was shown by the quality score assessment results in Table II.

In conclusion, this meta-analysis indicated that the -112G/A polymorphism of the UGRP1 gene may be involved in asthma, particularly in Asian populations. In the future, more well-designed, high-quality studies are required to assess the role of the UGRP1-112G/A polymorphisms in the pathogenesis of asthma.

## Figures and Tables

**Table I:** Search strategies.

Database	Time span	Search strategies
Pubmed	1966-March 12, 2013	((“SCGB3A2”[MH] → “SCGB3A2 protein, human[supplementary concept]”) OR(“SCG3A2”[ALL] OR “Uteroglobin-related protein1”[ALL] OR “Clara cell secretory protein-related protein”[ALL] OR “UGRP1”[ALL])) AND ((“polymorphism, genetic”[MH] OR “polymorphism, single nucleotide”[MH] OR (“polymorphism*”[ALL] OR “variant*”[ALL] OR “genetic*”[ALL] OR “mutant*” [ALL])) AND (“asthma”[MH] OR “asthma”[ALL] OR “bronchial hyperreactivity”[ALL] OR “respiratory hypersensitivity” [ALL] OR “bronchial asthma*”[ALL]) AND “humans”[MH]
EBSCOhost	1997-March 8, 2013	(asthma[subject terms] OR asthma[all text] OR “bronchial asthma”[all text] OR “bronchial hyperreactivity”[all text] OR “respiratory hypersensitivity”[all text]) AND (polymorphism[subject terms] OR polymorphism[all text] OR “genetic polymorphism”[all text] OR “single nucleotide polymorphism”[all text] OR variant*[all text] OR mutant*[all text]) AND (SCG3A2[all text] OR “Uteroglobin-related protein1”[all text] OR “Clara cell secretory protein-related protein” [all text])
BIOSIS Previews	1950-March 12, 2013	#1. Topic=(UGRP1) OR Topic=(SCGB3A2) OR Topic=(Uteroglobin-related protein1) OR Topic=(Clara cell secretory protein-related protein) #2. Topic=(polymorphism*) OR Topic=(variant*) OR Topic=(mutant*) OR Topic=(genetic*) #3. Topic=(asthma) OR Topic=(bronchial asthma) OR Topic=(bronchial hyperreactivity) OR Topic=(respiratory hypersensitivity) #4. #1 AND #2 AND #3

SCGB3A2, secretoglobin, family 3A, member 2.

**Table II:** Characteristics of the seven case-control studies included in the meta-analysis.

						Genotype AA/GA/GG						
											
First author	Year	Country	Ethnicity	Age group	Atopic status	Case, n	Control, n	Asthma definition	Atopic definition	Genotyping method	HWE	Quality scores
Batra J	2005	India	Asian	Adults	Atopic	4/40/121	2/40/118	ATS diagnosis criteria	History	Sequencing	Yes	9
Heinzmann A	2003	Germany	Caucasian	Children	NA	5/38/139	7/63/169	Asthmatic symptoms, medication and bronchial hyperreactivity	SPT, specific and total IgE	PCR-RFLP	Yes	10
Inoue K	2008	Japan	Asian	Adults	Mixed^b^ 77.3%	12/53/76	5/32/66	Symptoms, FEV1 or PEFR, the absence of any other pulmonary diseases	Antigen-specific IgE or a non-specific IgE	RT-PCR	Yes	12
Jian Z	2003	Japan	Asian	Children Mixed^a^	NA	11/42/78	15/109/224	NA	NA	PCR-RFLP	Yes	11
Niimi T	2002	Japan	Asian	Adults	Mixed^b^ 67.9%	3/31/50	2/13/70	Symptoms, FEV1 or PEFR, airway hyperresponsiveness	NA	Sequencing	Yes	10
Rigoli L	2007	Italy	Caucasian	Children Mixed^a^	Atopic	9/30/74	10/67/153	Signs or symptoms were present	i) SPT, ii) specific IgE, iii) total IgE	PCR-RFLP	Yes	10

a Case group was children and the data for children and adults were able to be separately extracted in the control group;

b data for patients with atopic or non-atopic asthma were not able to be separately extracted.

SPT, skin prick test; Ig, immunoglobulin; FEV1, forced expiratory volume in 1 sec; PEFR, peak expiratory flow rate; ATS, American Thoracic Society; PCR, polymerase chain reaction; RFLP, restriction fragment length polymorphism; NA, not available; HWE, Hardy-Weinberg equilibrium.

**Table III:** Results of the pooled and subgroup analyses of the included studies for the association between the UGRP1 -112G/A polymorphism and asthma risk.

			A vs. G		AA vs. GG		AA/GA vs. GG		AA vs. GA/GG	
						
Variables	n	Cases/controls, n	OR (95% CI)	P_Eff_	OR (95% CI)	P_Eff_	OR (95% CI)	P_Eff_	OR (95% CI)	P_Eff_
Overall	6	816/1,165	1.24 (0.95–1.61)	0.11	1.76 (1.12–2.78)	**0.01**	0.81 (0.59–1.13)	0.22	1.70 (1.09–2.67)	**0.02**
Subgroup by ethnicity										
Asian	4	521/696	1.42 (1.06–1.90)	**0.02**	2.08 (1.16–3.72)	**0.01**	0.68 (0.45–1.02)	0.06	1.90 (1.07–3.38)	**0.03**
Caucasian	2	295/469	0.95 (0.66–1.39)	0.81	1.40 (0.68–2.92)	0.41	1.14 (0.82–1.59)	0.42	1.43 (0.69–2.94)	0.33
Subgroup by atopy										
Atopic	2	278/390	1.12 (0.83–1.50)	0.47	1.88 (0.82–4.30)	0.13	0.97 (0.69–1.36)	0.84	1.92 (0.85–4.35)	0.12
Mixed	2	225/188	1.84 (1.08–3.13)	**0.02**	2.09 (0.82–5.34)	0.12	0.47 (0.23–0.97)	**0.04**	1.75 (0.69–4.40)	0.24
NA	2	313/587	1.02 (0.63–1.66)	0.93	1.54 (0.79–3.00)	0.21	1.04 (0.64–1.69)	0.88	1.55 (0.80–3.01)	0.19

UGRP1, uteroglobin-related protein 1 gene; NA, not available; N, number of studies; OR, odds ratio; CI, confidence interval; P_Eff_, P-value of pooled effect; Mixed, data for atopic or non-atopic asthma patients were not able to be separately extracted.
